# Left atrial appendage closure in patients with chronic kidney disease: results from the German multicentre LAARGE registry

**DOI:** 10.1007/s00392-020-01638-5

**Published:** 2020-04-15

**Authors:** Christian Fastner, Johannes Brachmann, Thorsten Lewalter, Uwe Zeymer, Horst Sievert, Martin Borggrefe, Christoph A. Nienaber, Christian Weiß, Sven T. Pleger, Hüseyin Ince, Jens Maier, Stephan Achenbach, Holger H. Sigusch, Matthias Hochadel, Steffen Schneider, Jochen Senges, Ibrahim Akin

**Affiliations:** 1 First Department of Medicine, University Medical Centre Mannheim (UMM), Faculty of Medicine Mannheim, University of Heidelberg, European Center for AngioScience (ECAS), and DZHK (German Center for Cardiovascular Research) Partner Site Heidelberg/Mannheim, Theodor-Kutzer-Ufer 1-3, 68167 Mannheim, Germany; 2 Department of Cardiology, Angiology, and Pneumology, Second Medical Clinic, Coburg Hospital, Coburg, Germany; 3 Department of Medicine, Cardiology, and Intensive Care, Hospital Munich-Thalkirchen, Munich, Germany; 4 Klinikum Ludwigshafen, Ludwigshafen am Rhein, Germany; 5 CardioVascular Center (CVC) Frankfurt, Frankfurt, Germany; 6 Anglia Ruskin University, Chelmsford, UK; 7 Cardiology and Aortic Center, Royal Brompton and Harefield NHS Foundation, Trust at Imperial College, London, UK; 8 Department of Cardiology, Klinikum Lüneburg, Lüneburg, Germany; 9 Department of Internal Medicine III, Cardiology, Angiology, and Pneumology, University Hospital Heidelberg, Heidelberg, Germany; 10 Department of Cardiology, Rostock University Medical Center, Rostock, Germany; 11 Department of Cardiology, Vivantes Klinikum Am Urban & im Friedrichshain, Berlin, Germany; 12 Medical Department I, SLK-Kliniken Heilbronn GmbH, Klinikum am Gesundbrunnen, Heilbronn, Germany; 13 Department of Medicine, University of Erlangen, Erlangen, Germany; 14 Clinic for Internal Medicine I, Heinrich-Braun-Klinikum Zwickau gGmbH, Zwickau, Deutschland; 15 Stiftung Institut für Herzinfarktforschung, Ludwigshafen am Rhein, Germany

**Keywords:** Atrial fibrillation, Chronic kidney disease, Left atrial appendage, Left atrial appendage closure, LAARGE

## Abstract

**Objectives:**

Chronic kidney disease (CKD) is associated with an increased complication rate after cardiac interventions. Although CKD has a high prevalence among atrial fibrillation patients, the impact of CKD on periprocedural complications and the outcome after an interventional left atrial appendage closure (LAAC) is unclear. The present study, therefore, aimed to investigate whether CKD influences the procedure’s effectiveness and safety.

**Methods:**

LAARGE is a prospective, non-randomised registry. LAAC was conducted with different standard commercial devices, and the follow-up period was one year. CKD was defined by an eGFR < 60 mL/min/1.73 m^2^, and subgroups were further analysed (i.e. eGFR < 15, 15–29, and 30–59 mL/min/1.73 m^2^, respectively).

**Results:**

Two hundred ninety-nine of 623 patients (48.0%) revealed a CKD. The prevalence of cardiovascular comorbidity, CHA_2_DS_2_-VASc score (4.9 vs. 4.2), and HAS-BLED score (4.3 vs. 3.5) was significantly higher in CKD patients (each *p* < 0.001). Implantation success was similarly high across all GFR groups (97.9%). Periprocedural MACCE (0.7 vs. 0.3%), and other major complications (4.7 vs. 3.7%) were comparably infrequent. Survival free of stroke was significantly lower among CKD patients within 1 year (82.0 vs. 93.0%; *p* < 0.001; consistent after adjustment for confounding factors), without significant accentuation in advanced CKD (i.e. eGFR < 30 mL/min/1.73 m^2^; *p* > 0.05  vs. eGFR 30–59 mL/min/1.73 m^2^). Non-fatal strokes were absolutely infrequent during follow-up (0 vs. 1.1%). Severe non-fatal bleedings were observed only among CKD patients (1.4 vs. 0%; *p* = 0.021).

**Conclusions:**

Despite an increased cardiovascular risk profile of CKD patients, device implantation was safe, and LAAC was associated with effective stroke prevention across all CKD stages.

**Electronic supplementary material:**

The online version of this article (10.1007/s00392-020-01638-5) contains supplementary material, which is available to authorized users.

## Introduction

Stroke and systemic embolisation are prognostically relevant complications of atrial fibrillation (AF) [1]. In patients with non-valvular AF, > 90% of thrombi originate from the left atrial appendage (LAA), which is located in front of the left atrium, and has intensively trabeculated walls [2]. While the use of non-vitamin K antagonist oral anticoagulants (NOAC) is the recommended standard for prophylaxis in patients with non-valvular AF and a high thromboembolic risk [1], some patients reveal contraindications for a long-term use of such substances [3, 4]. For these patients, the left atrial appendage closure (LAAC) has evolved as an interventional alternative and was proven to be effective and safe in high-risk patients even without a post-procedural period with continued anticoagulation [1, 5].

While the prevalence of AF is high among patients with impaired renal function, these patients are prone to an increased thromboembolic risk compared to AF patients with normal renal function [6, 7]. Clinically relevant chronic kidney disease (CKD) is defined by an estimated glomerular filtration rate (eGFR) < 60 mL/min/1.73 m^2^ [8]. Besides the increased thromboembolic risk, bleeding complications are more frequent in patients with AF and concomitant CKD, particularly in patients who are anticoagulated [6]. This is also depicted by the integration of an impaired renal function as a risk factor in the HAS-BLED score [9]. Moreover, the use of NOACs should be avoided in patients with severely impaired renal function, i.e. eGFR < 15 mL/min/1.73 m^2^, because insufficient data are available, and warfarin use is associated with conflicting outcome results [1].

Especially in combination with other cardiovascular risk factors, CKD might render AF patients ineligible for long-term OAC and might favour LAAC in many of these patients. However, renal failure was also shown to increase the rate of periprocedural complications in cardiac interventions and to worsen the outcome [10, 11]. Currently, outcome data on LAAC in CKD patients are limited [12]. The present subanalysis of the Left-Atrium-Appendage occluder Register—GErmany (LAARGE, ClinicalTrials.gov Identifier: NCT02230748), therefore, aimed to investigate on the intra-hospital outcome as well as the effectiveness and safety during one-year follow-up in patients with AF and CKD.

## Methods

### The Registry

LAARGE is a prospective, non-randomised, multicentre real-world registry that encompasses patients from 37 voluntary participating centres. Its main objective is to represent the LAAC procedure’s clinical reality. For this reason, the protocol neither influenced indication nor clinical management, but it claimed consecutive enrolment to avoid a recruitment bias. Devices should be implanted according to current recommendations. Recruitment in the registry started in July 2014 and ended in January 2016.

For the present subanalysis, patients with started procedure and documented renal function were selected from the whole database. The study was carried out according to the principles of the declaration of Helsinki and was approved by the ethics committee of the State Chamber of Medicine in Rhineland-Palatinate, Germany. Written informed consent was obtained from all study patients.

### Definition of chronic kidney disease

The eGFR was calculated using The Modification of Diet in Renal Disease (MDRD) Study formula [8]. According to the guidelines, patients with eGFR < 60 mL/min/1.73 m^2^ were categorised as having a clinical relevant impaired renal function [13]. Patients with CKD were categorised into three groups (i.e. eGFR < 15 mL/min/1.73 m^2^, eGFR 15–29 mL/min/1.73 m^2^, and 30–59 mL/min/1.73 m^2^).

### Procedure

As described previously [14], the preprocedural screening, the conduction of the implantation procedure as well as the postprocedural management including the antithrombotic treatment were at the discretion of the operating physician. Different standard commercial devices were implanted taking into consideration the specific manufacturer’s recommendations.

### Data acquisition

All participating centres reported procedural data and intra-hospital complications as well as discharge medication via an electronic case report form. Patients were contacted directly or via phone call one year after the implantation procedure to assess the survival, the occurrence of complications, and the antithrombotic treatment. If no contact could be established with a patient, information was obtained from the registration offices. For the purpose of data validation, all relevant events were reviewed and evaluated by an Endpoint Adjudication Committee, if necessary based on the original medical documents.

### Outcome measures

The effectiveness was primarily assessed by the absence of all-cause death and stroke during follow-up, secondarily by the absence of transient ischemic attacks (TIA) and systemic embolism. The implantation success was defined as a stable device anchorage. Complications including para-device leaks > 5 mm, device dislocations, severe and moderate bleedings during hospitalisation and during follow-up as well as thromboembolism in the venous system represented the safety outcome measure.

### Statistics

Statistical analyses were performed with SAS^®^ version 9.4 (SAS Institute, Cary, NC, USA). Continuous data are presented as means with standard deviation or as medians with interquartile ranges (25th and 75th percentiles), categorical data as frequencies with group-related percentages. Trends across the patient groups were assessed by a Cochran-Armitage test regarding categorical variables, or by an exact Cochran-Armitage test in case of rare events, and by a Jonckheere–Terpstra test regarding metrical variables, as indicated in the tables. In addition, CKD patients were compared to non-CKD patients using the Pearson Chi-squared test or Mann–Whitney–Wilcoxon test for categorical and metrical variables, respectively. These statistics were based on the available cases.

The one-year mortality after the implantation procedure and the incidence of the combined event of death or stroke were evaluated by methods of survival analysis (Kaplan–Meier curves, log-rank test). Hazard ratios with 95% confidence intervals (CI) were estimated using Cox regression without adjustment and adjusted for baseline characteristics significantly associated with CKD and known as clinically relevant risk factors: age (linear), sex, body mass index > 25 kg/m^2^, arterial hypertension, diabetes mellitus, coronary artery disease, congestive heart failure, and LVEF ≤ 40%. The expected annual rates of major bleeding and stroke were calculated from the individual HAS-BLED [9] and CHA_2_DS_2_-VASc score, respectively [15]. The follow-up duration was defined as the time span from the index discharge to the date of the follow-up contact. *p *values ≤ 0.05 (two-tailed) were considered significant.

## Results

### Baseline characteristics

623 patients were included in the present analysis. 299 (48.0%) revealed a CKD (Table 1). The median eGFR value was calculated at 41.1 vs. 78.8 mL/min/1.73 m^3^ (*p* < 0.001 for the comparison to non-CKD patients). CKD patients were significantly older (77.8 ± 7.5 vs. 74.4 ± 7.8 years, *p* < 0.001) and revealed a significantly higher stroke (CHA_2_DS_2_-VASc score 4.9 ± 1.5 vs. 4.2 ± 1.5, *p* < 0.001) and bleeding risk (HAS-BLED score 4.3 ± 1.0 vs. 3.5 ± 1.0, *p* < 0.001), whereby an HAS-BLED score ≥ 3, corresponding to a high bleeding risk, was significantly more frequent in CKD patients (97.6 vs. 84.7%, *p* < 0.001). CKD patients also revealed a more pronounced cardiovascular risk profile.

**Table 1:** Baseline characteristics

	eGFR < 15 mL/min	eGFR 15–29 mL/min	eGFR 30–59 mL/min	No CKD	*p* value for trend*
Total cohort, *n* (% of all patients)	15 (2.4)	45 (7.2)	239 (38.4)	324 (52.0)	
Male, *n* (%)	12 (80.0)	26 (57.8)	124 (51.9)	218 (67.3)	0.069
Age [years], median (IQR)	75 (69; 79)	80 (76; 82)	79 (74; 83)	76 (71; 80)	** < 0.001**
Body mass index [kg/m^2^], median (IQR)	25 (23; 32)	28 (25; 30)	27 (24; 31)	26 (24; 30)	**0.038**
CHA_2_DS_2_-VASc score, mean ± SD	5.1 ± 1.7	5.3 ± 1.6	4.8 ± 1.4	4.2 ± 1.5	** < 0.001**
HAS-BLED score, mean ± SD	4.6 ± 1.1	4.8 ± 0.9	4.2 ± 1.0	3.5 ± 1.0	** < 0.001**
Type of AF, each *n* (%)					
Paroxysmal	7 (46.7)	16 (35.6)	99 (41.4)	144 (44.4)	0.39
Persistent	3 (20.0)	10 (22.2)	42 (17.6)	57 (17.6)	0.59
Permanent	5 (33.3)	19 (42.2)	98 (41.0)	123 (38.0)	0.66
Congestive heart failure, *n* (%)	6 (40.0)	22 (48.9)	74 (31.0)	69 (21.3)	** < 0.001**
Arterial hypertension, *n* (%)	14 (93.3)	43 (95.6)	222 (92.9)	301 (92.9)	0.69
Diabetes mellitus, *n* (%)	10 (66.7)	24 (53.3)	96 (40.2)	84 (25.9)	** < 0.001**
Prior cerebrovascular event, each *n* (%)					
TIA	1 (6.7)	4 (8.9)	14 (5.9)	33 (10.2)	0.22
Stroke	3 (20.0)	11 (24.4)	46 (19.2)	72 (22.2)	0.72
Coronary heart disease, *n* (%)	11 (73.3)	22 (48.9)	133 (55.6)	123 (38.0)	** < 0.001**
Prior CABG, *n* (%)	2 (13.3)	7 (15.6)	35 (14.6)	29 (9.0)	0.056
Peripheral arterial disease, *n* (%)	6 (40.0)	17 (37.8)	66 (27.6)	74 (22.8)	**0.012**
Prior major bleeding, *n* (%)	6 (40.0)	18 (40.0)	94 (39.3)	131 (40.4)	0.87
Indication for LAAC, each *n* (%)					
Prior bleeding	14 (93.3)	37 (82.2)	199 (83.3)	247 (76.2)	**0.022**
Prior cerebrovascular event despite OAC	4 (26.7)	13 (28.9)	52 (21.8)	98 (30.2)	0.2
Absolute contraindication against any OAC	3 (20.0)	7 (15.6)	48 (20.1)	62 (19.1)	0.88
Labile INR	1 (6.7)	6 (13.3)	27 (11.3)	20 (6.2)	0.061
Incompliance with OAC	0 (0.0)	5 (11.1)	15 (6.3)	13 (4.0)	0.2
Patient preference	3 (20.0)	5 (11.1)	54 (22.6)	88 (27.2)	**0.028**
Other reason	2 (13.3)	5 (11.1)	20 (8.4)	31 (9.6)	0.82
Medication at presentation, each *n* (%)					
Anticoagulants	9 (60.0)	27 (60.0)	153 (64.0)	193 (59.6)	0.6
Antiplatelet agent	6 (40.0)	19 (42.2)	92 (38.5)	102 (31.5)	0.056

*AF* atrial fibrillation, *CKD* chronic kidney disease, *eGFR* estimated glomerular filtration rate, *INR* international normalised ratio, *IQR* interquartile range,   *LAAC* left atrial appendage closure,   *MDRD* modification of diet in renal disease,     *OAC* oral anticoagulation, *SD* standard deviation, *TIA* transitory ischemic attack

^*^Tested by Cochran–Armitage or Jonckheere–Terpstra test; *p* < 0.05 is indicating a significant difference (printed in bold type)

Participating centres could document more than one indication for LAAC in the same patient (Table 1). Across all predefined GFR groups, the main indication was a prior bleeding event (79.8%; *p* = 0.022 for trend).

Supplemental Table 1 shows data from cardiac imaging procedures. While left atrial diameters were larger in CKD patients (*p* = 0.024 for trend), this finding did not correspond with the LAA diameters (each *p* > 0.05 for trend).

### Procedural data and intra-hospital outcome

Technical success was high across all groups (97.9%; *p* = 0.76 for trend; supplemental Table 2), and no peri-device leak > 5 mm was present. Three interventions had to be interrupted prematurely (*p *= 0.87 for trend). A stable device anchorage could not be achieved in additional three patients. Device selection and dimensions as well as procedural parameters did not differ significantly (each *p* > 0.05 for trend).

**Table 2:** Intra-hospital outcome

	eGFR < 15 mL/min	eGFR 15–29 mL/min	eGFR 30–59 mL/min	No CKD	*p* value for trend*
Total cohort, *n* (% of all patients)	15 (2.4)	45 (7.2)	239 (38.4)	324 (52.0)	
MACCE, *n* (%)	0 (0.0)	2 (4.4)	0 (0.0)	1 (0.3)	0.097
Death, *n* (%)	0 (0.0)	2 (4.4)	0 (0.0)	0 (0.0)	**0.028**
Myocardial infarction, *n* (%)	0 (0.0)	0 (0.0)	0 (0.0)	1 (0.3)	0.62
Stroke, *n* (%)	0 (0.0)	0 (0.0)	0 (0.0)	1 (0.3)	0.62
Other major complication, *n* (%)	2 (13.3)	2 (4.4)	10 (4.2)	12 (3.7)	0.27
Severe bleeding, *n* (%)	1 (6.7)	0 (0.0)	3 (1.3)	3 (0.9)	0.43
AV fistula or pseudoaneurysm, *n* (%)	0 (0.0)	1 (2.2)	2 (0.8)	3 (0.9)	1.0
Pericardial effusion requiring action, each *n* (%)					
Surgery	0 (0.0)	0 (0.0)	0 (0.0)	2 (0.6)	0.38
Intervention	1 (6.7)	1 (2.2)	5 (2.1)	6 (1.9)	0.44
Device dislodgement requiring action, each *n* (%)					
Surgery	0 (0.0)	0 (0.0)	0 (0.0)	0 (0.0)	–
Additional intervention	1 (6.7)	0 (0.0)	1 (0.4)	0 (0.0)	**0.028**
Moderate complications, *n* (%)	4 (26.7)	5 (11.1)	20 (8.4)	29 (9.0)	0.18
Moderate bleeding, *n* (%)	1 (6.7)	2 (4.4)	2 (0.8)	6 (1.9)	0.4
TIA, *n* (%)	0 (0.0)	0 (0.0)	0 (0.0)	0 (0.0)	–
Successful cardiopulmonary resuscitation, *n* (%)	0 (0.0)	0 (0.0)	1 (0.4)	2 (0.6)	0.71
Access site infection, *n* (%)	0 (0.0)	0 (0.0)	1 (0.4)	0 (0.0)	1.0
Pericardial effusion with conservative treatment, *n* (%)	0 (0.0)	0 (0.0)	4 (1.7)	7 (2.2)	0.31
Device dislodgement handled by immediate retraction, *n* (%)	0 (0.0)	0 (0.0)	3 (1.3)	2 (0.6)	1.0

*AV* arteriovenous, *CKD* chronic kidney disease, *eGFR* estimated glomerular filtration rate, *MACCE* major adverse cardiac and cerebrovascular events, *TIA* transitory ischemic attack

^*^Tested by exact Cochran–Armitage test; *p* < 0.05 is indicating a significant difference (printed in bold type)

Intra-hospital complications, and particularly major adverse and cerebrovascular events (MACCE) were generally rare (each *p* > 0.05 for trend; Table 2). Correspondingly, time to discharge was generally short (*p* = 0.097 for trend). Two intra-hospital deaths among the CKD patients were due to either an unknown or of cardiovascular aetiology, respectively. Seven dislodged devices could be retrieved catheter-based (each *p* > 0.05 for trend). Antithrombotic discharge medication did not differ significantly (each *p* > 0.05 for trend; supplemental Fig. 1), provided that 12.2% of patients stayed on anticoagulation when leaving the hospital (*p* = 0.57 for trend).

**Fig. 1:**
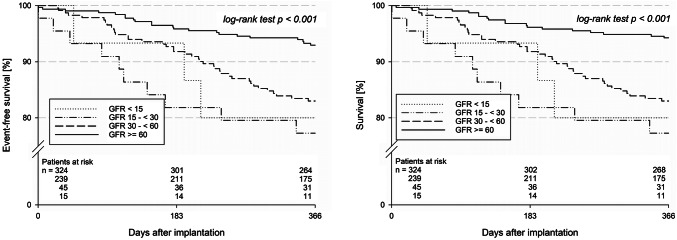
One-year incidence of all-cause death and stroke after left atrial appendage closure (LAAC). Left figure: freedom from all-cause death and stroke after left atrial appendage closure; right figure: freedom from all-cause death after LAAC

### Follow-up

**Fig. 2:**
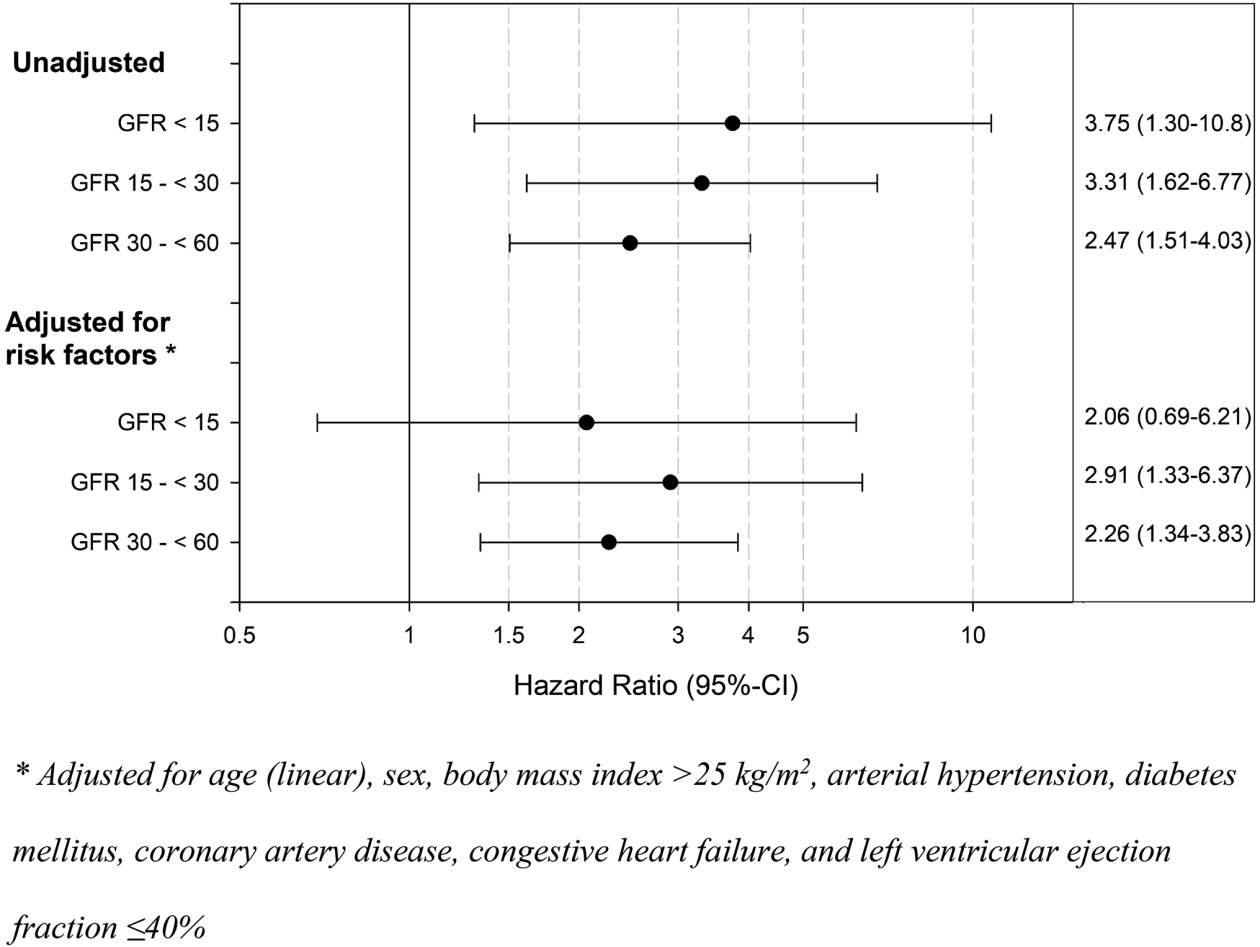
Adjustment of the primary efficacy outcome measure for relevant risk factors

A total of 608 patients (97.9%) could be followed-up (*p* = 0.85 for trend; Table 3). Limited to 365 days after the procedure, the combined primary effectiveness outcome measure was reached in 82.0% among CKD patients and 93.0% among patients without impaired renal function (*p* < 0.001; Fig. 1). Even after adjustment for relevant risk factors, this effect was still present (Fig. 2), but there was no statistically significant difference when comparing the patient groups with an eGFR < 60 mL/min/1.73 m^2^ among each other (*p* = 0.76). Only three non-fatal strokes were observed in the total cohort (*p* = 0.25 for trend), which all were ischemic. Moreover, rates of TIA and systemic embolism were low cross all GFR groups (each *p* > 0.05 for trend). Severe (*p* = 0.021 for trend) and moderate bleedings (*p* = 0.52 for trend) were infrequent across all groups. Despite only 6.0% of patients received anticoagulation after one year (*p* =  0.13 for trend), only two deep vein thromboses were registered (*p* = 1.00 for trend). 89.6% of patients were completely content with the intervention, and 96.6% of patients felt safe during hospital stay (each *p* > 0.05 for trend).

**Table 3:** Follow-up data

	eGFR < 15 mL/min	eGFR 15–29 mL/min	eGFR 30–59 mL/min	No CKD	*p* value for trend*
Discharged alive, *n*	15	43	239	324	
Documented follow-up, *n* (%)	15 (100.0)	42 (97.7)	234 (97.9)	317 (97.8)	0.85
Death, *n* (% of patients with documented vital status)	3 (20.0)	8 (19.0)	39 (16.7)	18 (5.7)	**< 0.001**
Events in survivors of total follow-up					
Stroke, *n* (%)	0 (0.0)	0 (0.0)	0 (0.0)	3 (1.1)	0.25
TIA, *n* (%)	0 (0.0)	0 (0.0)	1 (0.6)	1 (0.4)	1.0
Systemic embolism, *n* (%)	0 (0.0)	1 (3.4)	0 (0.0)	0 (0.0)	0.08
Major adverse events					
Device dislodgement requiring action, each *n* (%)					
Surgery	0 (0.0)	1 (3.6)	0 (0.0)	2 (0.7)	1.0
Additional intervention	0 (0.0)	0 (0.0)	1 (0.6)	0 (0.0)	1.0
Pericardial effusion requiring action, each *n* (%)					
Surgery	0 (0.0)	0 (0.0)	0 (0.0)	0 (0.0)	–
Intervention	0 (0.0)	0 (0.0)	0 (0.0)	1 (0.4)	0.64
Pulmonary embolism, *n* (%)	0 (0.0)	1 (3.4)	5 (2.8)	0 (0.0)	**0.04**
Severe bleeding, *n* (%)	1 (9.1)	0 (0.0)	2 (1.1)	0 (0.0)	**0.021**
Moderate adverse events					
Deep vein thrombosis, *n* (%)	0 (0.0)	0 (0.0)	1 (0.6)	1 (0.4)	1.0
Moderate bleeding, *n* (%)	1 (9.1)	1 (3.4)	8 (4.5)	10 (3.6)	0.52
Antithrombotic medication, each *n* (%)					
Anticoagulants	1 (9.1)	4 (13.8)	11 (6.1)	14 (5.1)	0.13
Antiplatelet agents	8 (72.7)	27 (93.1)	152 (84.9)	232 (83.8)	0.74
Subjective feeling of treatment success, each *n* (%)					
Completely	7 (87.5)	23 (92.0)	141 (91.0)	210 (88.6)	0.53
Partly	1 (12.5)	1 (4.0)	8 (5.2)	16 (6.8)	0.77
Not	0 (0.0)	1 (4.0)	4 (2.6)	11 (4.6)	0.33
Subjective feeling of safety during index hospitalisation, *n* (%)	9 (100.0)	25 (96.2)	153 (96.2)	237 (96.7)	0.96

*CKD* chronic kidney disease, *eGFR* estimated glomerular filtration rate, *TIA* transitory ischemic attack

^*^Tested by exact Cochran–Armitage (events) or asymptotic Cochran–Armitage test; *p* < 0.05 is indicating a significant difference (printed in bold type)

## Discussion

This subanalysis of the multicentre LAARGE registry confirmed an excellent procedural success and could demonstrate that LAAC was associated with effective stroke prevention also in patients with CKD.

Almost half of the patients (48.0%) were affected by a renal impairment. Despite an accentuated cardiovascular risk profile of CKD patients, and in contrast to prior published data [6, 7], patients were affected by a similar number of prior strokes across all stages. CKD patients are also known to be at higher risk for bleeding independent from the use of OAC [6, 7]. In our analysis, this was reflected by significantly more patients in the CKD group who were indicated for LAAC due to prior bleedings.

Independent from the renal function, the implantation success was high (97.9%). Periprocedural MACCE and other major complications were infrequent in both, patients with and without renal impairment, and rates were comparable to other recently published data [16]. This observation differentiates the LAAC procedure from interventions in the arterial system, as these cardiac procedures were shown to be associated with higher periprocedural complication rates and a worse outcome in CKD patients [10, 11, 17]. A fact which might be explained by intra-arterial administration being an independent risk factor for contrast-induced acute kidney injury [18]. Such low complication rates in contrast with the initial PROTECT-AF trial, reporting 8.9% of major adverse events, might also reflect the growing experience with the LAAC procedure [19].

Even after adjustment for relevant risk factors, the combined incidence of all-cause death and stroke was higher in the CKD group during follow-up, but was not accentuated in patients with an advanced renal insufficiency (i.e. eGFR < 30 mL/min/1.73 m^2^). Cases of death accounted for the vast majority of all these events (100 and 85.7%, respectively). An excess mortality among renally impaired patients is certainly not unexpected in a patient collective that is prone to a pre-existing and well described higher baseline risk.

Despite an increased risk of thromboembolic events, as reflected by a CHA_2_DS_2_-VASc score of 4.9 vs. 4.2, and thus despite a collective at noticeably higher risk than in the initial trials [19, 20], non-fatal strokes were extremely infrequent in both, patients with and without renal impairment (0 vs. 1.1%, respectively), standing for a dramatic reduction compared to the estimated annual stroke risk of 6.3 and 5.3%, respectively [15]. By stating that the majority of patients would otherwise not have been anticoagulated, this is a remarkable result, in particular in the more vulnerable CKD patients who comparably benefited.

The observed annual major bleeding rate was low, too, but, nonetheless, all major bleedings appeared in CKD patients. A finding which is not surprising given frequent analogous reporting in literature [6]. Against such a backcloth, it is all the more remarkable that the observed rates were much lower than the expected annual major bleeding rates based on the HAS-BLED score of 9.2 and 6.4%, respectively [9]. Moderate bleedings were infrequent across all stages of renal function. Despite only 6.0% of patients who were anticoagulated after one year, only 3.2 and 0.4% of patients suffered a thromboembolic event in the venous system. Thus, the LAAC procedure was shown to be a safe alternative for AF patients with renal impairment, while NOACs, which are also recommended for this subpopulation [1, 21], are associated with conflicting safety results particularly concerning bleeding events in CKD patients [21, 22].

These achievements may have contributed substantially to the fact that the intervened patients were highly content with the procedure (91.0 vs. 88.6%) and felt safe during the index hospitalisation (96.4 vs. 96.7%), which highlights the not only theoretical but also practical impact on the quality of life.

### Study limitations

These analyses were based on observational registry data with the inherent limitations of this study type. The conduction of the intervention was not influenced by the study protocol and based on the operators’ discretions as well as the relevant recommendations, which respected the observational character of the registry. This individualised decision algorithm may have had impact on the outcome measures but surely reflects the clinical practice. The implantation volume per centre and per operator was naturally heterogeneous, which also meant a good mixture of experience. Though a separate group with renal replacement therapy was envisaged, there were not enough cases to perform an individual analysis on such patients. Despite these limitations, this registry is surely serving as a data source for a little-studied topic.

## Conclusions

Despite an increased cardiovascular risk profile of CKD patients, a consistently high implantation success with low complication rates was seen in all stages of renal function. The observed stroke rates were comparably low in all groups.

## Electronic supplementary material

Below is the link to the electronic supplementary material.

Supplementary file1 (DOCX 344 kb)

## Abbreviations

AF: Atrial fibrillation
CI: Confidence interval
CKD: Chronic kidney disease
eGFR: Estimated glomerular filtration rate
LAA: Left atrial appendage
LAAC: Left atrial appendage closure
LAARGE: Left-Atrium-Appendage occluder Register GErmany
MACCE: Major adverse cardiac and cerebrovascular events
MDRD: Modification of diet in renal disease
(N)OAC: (Non-vitaminK antagonist) oral anticoagulants
TIA: Transient ischemic attack
