# Supplementary material for: Left atrial appendage closure in patients with chronic kidney disease: results from the German multicentre LAARGE registry
Source: Clin Res Cardiol. 2020 Apr 15;110(1):12–20. doi: 10.1007/s00392-020-01638-5 (PMC7806558; doi:10.1007/s00392-020-01638-5)
Supplement: Supplementary file 1 — Supplementary file1 (DOCX 344 kb) [file 392_2020_1638_MOESM1_ESM.docx]

| **Supplemental Table 1: Results of the preinterventional cardiac imaging** | | | | | |
| --- | --- | --- | --- | --- | --- |
|  | **eGFR <1 5 mL/min** | **eGFR 15-29 mL/min** | **eGFR 30-59 mL/min** | **No CKD** | **p value for trend*** |
| **Total cohort, *n* (% of all patients)** | 15 (2.4) | 45 (7.2) | 239 (38.4) | 324 (52.0) |  |
| **LVEF ≤ 40%, *n* (%)** | 4 (26.7) | 8 (18.6) | 32 (13.7) | 29 (9.2) | **0.006** |
| **LA diameter [mm], median (IQR)** | 49 (45; 52) | 49 (45; 53) | 48 (44; 52) | 47 (43; 51) | **0.024** |
| **LAA ostial diameter [mm], median (IQR)**   - **0°** - **45°** - **90°** - **135°** | 22 (22; 24)  21 (19; 22)  21 (19; 24)  23 (21; 26) | 19 (17; 21)  20 (17; 22)  19 (16; 22)  19 (17; 22) | 20 (18; 22)  20 (18; 22)  20 (18; 22)  20 (18; 23) | 19 (18; 22)  20 (17; 22)  20 (17; 22)  20 (18; 22) | 0.093  0.45  0.25  0.53 |
| **LAA morphology, each *n* (%)**   - **Cactus** - **Cauliflower** - **Chicken wing** - **Windsock** - **Undefined** | 2 (13.3)  2 (13.3)  8 (53.3)  2 (13.3)  1 (6.7) | 7 (17.1)  6 (14.6)  16 (39.0)  7 (17.1)  5 (12.2) | 14 (6.2)  40 (17.8)  99 (44.0)  40 (17.8)  32 (14.2) | 30 (10.3)  40 (13.8)  133 (45.9)  40 (13.8)  47 (16.2) | 0.79  0.55  0.75  0.42  0.24 |
| * Tested by Cochran-Armitage or Jonckheere-Terpstra test; p<0.05 is indicating significant difference (printed in bold type); CKD = chronic kidney disease; eGFR = estimated glomerular filtration rate; IQR = interquartile range; LA = left atrial; LAA = left atrial appendage; LVEF = left ventricular ejection fraction | | | | | |

| **Supplemental Table 2: Procedural data** | | | | | |
| --- | --- | --- | --- | --- | --- |
|  | **eGFR < 15 mL/min** | **eGFR 15-29 mL/min** | **eGFR 30-59 mL/min** | **No CKD** | **p value for trend*** |
| **Total cohort, n (% of all patients)** | 15 (2.4) | 45 (7.2) | 239 (38.4) | 324 (52.0) |  |
| **Successful implantation, n (%)** | 15 (100.0) | 43 (95.6) | 236 (98.7) | 316 (97.5) | 0.76 |
| **Procedure interrupted, n (%)** | 0 (0.0) | 1 (2.2) | 0 (0.0) | 2 (0.6) | 0.87 |
| **Number of implantation attempts, mean±SD** | 1.7±1.3 | 1.7±1.6 | 1.7±1.3 | 1.5±1.1 | 0.10 |
| **Para-device leak, each n (%)**   - **< 3 mm** - **3-5 mm** - **> 5 mm** | 2 (13.3)  2 (100.0)  0 (0.0)  0 (0.0) | 1 (2.3)  1 (100.0)  0 (0.0)  0 (0.0) | 16 (6.8)  13 (81.3)  3 (18.8)  0 (0.0) | 13 (4.2)  8 (61.5)  5 (38.5)  0 (0.0) | 0.27 |
| **Type of LAAC device, each n (%)**   - **WATCHMAN™** - **AMPLATZER™ Cardiac Plug** - **AMPLATZER™ Amulet™** - **Other device** | 7 (46.7)  5 (33.3)  3 (20.0)  0 (0.0) | 22 (48.9)  16 (35.6)  7 (15.6)  0 (0.0) | 99 (41.4)  70 (29.3)  62 (25.9)  8 (3.3) | 145 (44.8)  82 (25.3)  87 (26.9)  10 (3.1) | 0.99  0.11  0.18  0.45 |
| **Total duration [min], median (IQR)** | 60 (40; 76) | 60 (43; 75) | 60 (42; 85) | 56 (43; 75) | 0.40 |
| **Fluoroscopy time [min], median (IQR)** | 10 (7; 12) | 10 (6; 13) | 10 (7; 15) | 11 (7; 15) | 0.37 |
| **Dose area product [cGy*cm^2^], median (IQR)** | 2725 (1172; 6498) | 1549 (611; 5796) | 2070 (843; 3674) | 1950 (795; 4204) | 0.94 |
| **Hospital stay after procedure [d], median (IQR)** | 3 (1; 9) | 2 (1; 4) | 2 (2; 4) | 2 (1; 3) | 0.097 |
| * Tested by Cochran-Armitage or Jonckheere-Terpstra test; p<0.05 is indicating significant difference; CKD = chronic kidney disease; eGFR = estimated glomerular filtration rate; IQR = interquartile range; LAAC = left atrial appendage closure; SD = standard deviation | | | | | |

**Supplemental Figure 1: Antithrombotic therapy**

*

*

*Pie charts characterising the antithrombotic treatment (AT) at discharge in patients with CKD (left) and without CKD (right); APT =antiplatelet therapy*
